# Undernutrition before two years in Algeria: Repeated cross-sectional analyses (2012-13 to 2018-19)

**DOI:** 10.12688/f1000research.158413.1

**Published:** 2024-11-26

**Authors:** Nagwa Farag Elmighrabi, Catharine A. K. Fleming, Kingsley E. Agho

**Affiliations:** 1 Faculty of Public Health, Department of Nutrition, University of Benghazi, Benghazi, Benghazi, 16063, Libya; 2 Organization of People of Determination and Sustainable Development, Benghazi, Benghazi district, Libya; 3 School Of Health Sciences, Campbelltown Campus, Western Sydney University, South Penrith, New South Wales, 1797, Australia; 4 Translational Health Research Institute (THRI), School of Medicine, Western Sydney University, Penrith, 2750, Australia; 5 Faculty of Health Sciences, University of Johannesburg, Johannesburg, 2094, South Africa

**Keywords:** Stunting, wasting, underweight, young children, North Africa

## Abstract

**Background:**

Algeria has made significant progress in addressing childhood undernutrition. Despite improvements, nearly 12% of Algerian children under five years of age suffered from stunting in 2022. This study aimed to evaluate the prevalence of three indicators of undernutrition (stunting, wasting, and underweight) and their associated variables in children aged 0–23 months in Algeria between 2013 and 2019.

**Methods:**

Pooled data from Multiple Indicator Cluster Surveys (MICS) (2012-13 and 2018-19) covering 14,498 children were used. Univariate and multiple logistic regression analyses were performed to test the associations.

**Results:**

Stunting decreased significantly, but wasting and being underweight showed no significant reductions from 2013 to 2019. Stunting, wasting, and underweight decreased by 2.2%, 1.3%, and 0.3%, respectively, with an overall prevalence of 10.1%, 5.4%, and 3.6%, respectively. Factors associated with undernutrition included child age and small size at birth, whereas stunting was linked to the poorest households, male children, higher maternal body mass index (BMI), and low antenatal care (ANC) visits. Wasting was associated with younger age and paternal age, fewer maternal ANC visits, underweight male sex, low maternal education, high maternal BMI, and delayed breastfeeding initiation.

**Conclusion:**

Public health experts and stakeholders must adopt a comprehensive community-centered approach to achieve long-term improvements in child nutrition in Algeria. This strategy should focus on key factors including high maternal BMI, low maternal education, male children, and infrequent ANC visits. This action can help Algeria progress towards achieving the UN’s goal of eradicating malnutrition.

## Background

Child undernutrition remains a major public health issue and one of the leading causes of morbidity and mortality worldwide. The World Health Organization (WHO) states that undernutrition accounts for nearly half of all deaths in children under five globally, which could be prevented through maternal and child health interventions. According to recent global estimates, there are 149 million stunted children under five worldwide, of whom 45 million have wasting and 14.3 million severe wasting. ^1^ Most undernourished children are in developing countries, with Asian and African countries being the most affected. In Asia, approximately 54% of children under five years of age are stunted, while 69% suffer from wasting. In Africa, approximately 40% of children under five experience stunting and 27% experience wasting. ^2^

The WHO has reported alarming statistics that show a worrying rise in undernutrition patterns in North Africa, which runs counter to the global trend according to its release in 2021. ^3^ In 2022, approximately 12% of children under the age of five in Algeria experienced stunting, while the prevalence of wasting in the country was 4.1% in the same year. Stunting is frequently observed in children who endure extended durations of insufficient nourishment, resulting in the depletion of vital nutrients necessary for growth. ^4^

The seriousness of undernutrition lies in its potential to cause permanent physical and cognitive impairments in children, especially in those who experience stunting. ^1, 2, 5^ The long-lasting consequences of stunting include decreased cognitive function, hindered academic performance, lower income in adulthood, reduced productivity, and an elevated risk of nutrition-related chronic diseases in the later stages of life. These effects have been observed throughout the developmental journey from adolescence to young adulthood. ^2, 6^ Insufficient nutrient intake and/or recurring illnesses can lead to wasting and pose a significant threat to life. ^1^ When children suffer from wasting, they become more vulnerable to long-term developmental delays, weakened immunity, and elevated risk of death. ^1^ Undernutrition not only increases the rates of child mortality and morbidity by endangering immunity and increasing susceptibility to infections, but also has broader socioeconomic ramifications and effects on family incomes, community prosperity, and productivity. ^2, 7^

This study specifically targeted children under two years of age, as evidence suggests that inadequate fetal growth and/or stunting during the first two years of life can result in irreversible effects, including shorter adult height, lower educational achievement, reduced income, and reduced birth weight of future offspring. Furthermore, children who experience undernourishment in their early years and subsequently experience rapid weight gain are susceptible to nutrition-related chronic illnesses. ^8^ The most critical time for nutrition interventions is before and/or during the first two years, as they greatly affect child survival, health, and development. ^9^

Previous studies have identified factors that influence undernutrition. ^7, 10^ Such factors include poverty, natural disasters, food insecurity, ^11, 12^ economic difficulties, civil wars, ^12^ climate change ^13^ and epidemics. ^14^ The more prevalent detrimental factors in middle-income countries have the potential to further worsen the immediate factors associated with undernutrition, particularly maternal health, ^15^ food intake, ^16^ and illnesses. ^17^ According to the findings of the Global Nutrition Report, Algeria has made considerable strides in its efforts to combat stunting. Nevertheless, it is important to highlight that 9.8% of children under the age of five are still affected, a figure that falls below the average for the African region (30.7%). Algeria is making progress toward achieving the target for wasting, as only 2.7% of children under five are affected, which is still lower than that in Africa (6.0%). ^18^

However, the country continues to grapple with the aforementioned factors, which collectively contribute to the perpetuation or exacerbation of malnutrition. Furthermore, the United Nations Economic and Social Commission for Western Asia (ESCWA) found that Algeria is currently grappling with a triple burden of malnutrition. The country has a high food import dependency with high costs, water scarcity, and high unemployment, which could further affect its food security. Food insecurity has emerged as a potent stressor for families with significant adverse effects on children’s health and development. ^19^

In 2020, a publication updated the nutritional status of Eastern Algeria. ^20^ In 2023, a systematic review and meta-analysis analyzed childhood undernutrition in North Africa, including Algeria, through a systematic review and meta-analysis study. ^21^ However, a significant limitation of these studies is the issue of generalizability, owing to the small number of undernourished children recorded in a single dataset. Additionally, including Algeria, along with other countries, in the second study may have led to an over- or underestimation of the findings. Consequently, this study combined the two datasets to enhance statistical power and ability to compare outcomes, and to overcome the inconclusive results from a single study. This study aimed to evaluate the presence of undernutrition (stunting, wasting, and underweight) and its associated factors among children aged 0–23 months in Algeria in 2013 and 2019. The results will enhance the existing body of evidence necessary for implementing effective interventions that prioritize actions targeting the factors associated with child malnutrition in Algeria.

## Methods

### Study design and setting

This cross-sectional study utilized nationally representative data from the Algerian Multiple Indicator Cluster Surveys (MICS) conducted in 2012-13 (as part of the fourth global iteration (MICS4)), and 2018-19 (as part of its sixth edition (MICS 2018-19)). Both surveys were implemented and facilitated by the Ministry of Health, Population, and Hospital Reform (Algeria) in collaboration with the global MICS program. These efforts have received financial and technical support from UNICEF, along with financial contributions from the United Nations Population Fund (UNFPA). ^22, 23^ The MICS gathers cross-sectional data internationally to provide comparable health information, especially regarding maternal and child health. The survey encompasses the entire nation, with an emphasis on particular regions. To achieve comparability, the MICS adopt a standardized complex sampling design that incorporated multistage stratified cluster sampling, and selection probabilities are assigned to each primary unit. The surveys are available at http://mics.unicef.org/.

### Study population

Eligible participants for data collection were all females aged 15–49 years who were either permanent residents or visitors residing in the households the night before the survey. A survey was conducted among mothers to collect data regarding the health of their youngest child, who was aged under five years. Country-specific MICS reports provide further details on the sampling method and questionnaire used. The response rate for nearly all women’s questionnaires ranged from 97% to 99%. In total, 14,498 children aged 0–23 months were included in the analysis, encompassing the two MICS surveys. The 2012-13 MICS included 7890 children (of ages 0-23 months) and the 2018-19 MICS included 6608 children.

### Data collection

A survey was conducted among women aged 15-49 utilizing a specific questionnaire for data collection. The questionnaire asked about the participants’ educational attainment, residential location, media exposure, pregnancy history, childhood mortality rates, breastfeeding and feeding practices, vaccination and illness history during childhood, marital status, sexual activity, employment status, marital background characteristics, awareness and behaviors concerning HIV/AIDS and other sexually transmitted infections, and maternal mortality.

### Outcome variables

Three indicators of undernutrition - stunting, wasting, and underweight - were used as the outcome variables in this study. The prevalence of each indicator was calculated using the 2006 WHO growth reference, which compares a child’s growth to that of a healthy child in the same age group or reference population. ^24^ This is represented as a deviation from the median in terms of standard deviations (SDs). Stunting was defined as height-for-age (HAZ) Z-score≤−2 SD; wasting as weight-for-height (WHZ) Z-score≤−2 SD of the median and underweight as weight-for-age (WAZ) Z-score <−2 SD of the median.

### Potential confounding factors

Potential confounding variables were chosen based on a previous study conducted in 35 low- and middle-income countries. ^25^ The covariates included place of residence (urban/rural), pooled household wealth index, child’s age and sex, parents’ age, parents’ educational attainment and nutritional health, number of household members, birth order, marital status and age, utilization of health care services, water and sanitation conditions, access to media, early initiation and duration of breastfeeding, perceived baby size, and child illness (diarrhea, fever, cough, and any infection within the two weeks preceding the survey).

Table 1 offers a detailed overview of the categorization of independent variables. To ensure better understanding, specific variables were further categorized as: The household wealth index was developed utilizing the “wscore” scores derived from the integrated datasets through the principal component’s statistical method. The combined household scores were categorized into five groups: poorest, poor, middle, rich, and richest. The lowest 20% of households represent the poorest group, whereas the highest 20% constitute the richest group. The available toilet systems comprise flush or pour-flush toilets connected to a piped sewer system, septic tank pit latrines, ventilated improved pit latrines, pit latrines with a slab, or composting toilets. ^26^ The definition of protected sources of drinking water encompasses household connections, public standpipes, boreholes, protected dug wells, protected springs, and rainwater collection. ^27^

**Table 1.** Characteristics of the study population (children 0-23 months) in Algeria for the two MICS surveys (2012-13 and 2018-19).

Characteristics	2012-13 (n=7440)	2018-19 (n= 6392)	2012-2019 (n= 13,832)
**Area of residence**			
Urban	4775 (60.5)	3768 (57.0)	8123 (58.7)
Rural	3115 (39.5)	2840 (43.0)	5710 (41.3)
**Wealth Index**			
Richest	1763 (22.4)	1420 (21.5)	3002 (21.7)
Fourth	1705 (21.6)	1174 (17.8)	2745 (19.85)
Middle	1611 (20.4)	1232 (18.6)	2742 (19.8)
Poorer	1517 (19.2)	1490 (22.5)	2870 (20.8)
Poorest	1293 (16.4)	1293 (19.6)	2474 (17.9)
**Sex of baby**			
Boy	4113 (52.1)	3395 (51.4)	7156 (51.7)
Girl	3777 (47.9)	3213 (48.6)	6677 (48.3)
**Child age (months)**			
0-5	2125 (26.9)	1572 (23.8)	3474 (25.1)
6-11	1976 (25.1)	1608 (24.3)	3446 (24.9)
12-17	1908 (24.2)	1817 (27.5)	3555 (25.7)
18-23	1880 (23.8)	1611 (24.4)	3357 (24.3)
**Mother's age (years)**			
15-19	572 (7.5)	302 (4.7)	844 (6.1)
20-34	4850 (63.4)	4135 (64.2)	8577 (62.0)
35-49	2227 (29.1)	1999 (31.1)	4034 (29.2)
**Father's age (years)**			
18-34	2153 (34.6)	7 (0.1)	2061 (14.9)
35-44	2881 (46.3)	4609 (82.2)	7164 (51.8)
45+	1193 (19.2)	994 (17.7)	2096 (15.2)
**Mother age at marriage (years)**			
≤ 18	471 (7.4)	299 (5.2)	742 (5.4)
>18	5903 (92.6)	5502 (94.8)	10901 (78.8)
**Marital status**			
Married	6231 (81.5)	5606 (87.1)	11320 (61.8)
Not married	1417 (18.5)	831 (12.9)	2135 (15.4)
**Polygamy status**			
No	6063 (97.5)	5486 (98.1)	11043 (79.83)
Yes	154 (2.5)	107 (1.9)	251 (1.8)
**Maternal education**			
Secondary and above	2825 (36.9)	2881 (44.8)	5443 (39.4)
Primary	3572 (46.7)	1972 (30.7)	5273 (38.1)
No education	1251 (16.4)	1581 (24.6)	2735 (19.8)
**Father education**			
Secondary and above	2767 (35.1)	2920 (44.2)	5419 (39.2)
Primary	3757 (47.6)	2041 (30.9)	5500 (39.8)
No education	1365 (17.3)	1647 (24.9)	2913 (21.1)
**Maternal BMI**			
≤18.5	5278 (71.1)	5654 (73.1)	9932 (71.8)
19-25	2095 (28.2)	1651 (25.9)	3746 (27.1)
25+	54 (0.7)	59 (0.9)	113 (8.0)
**Household members**			
1-3	873 (11.1)	1833 (27.7)	2578 (18.6)
4-8	4439 (56.3)	4248 (64.3)	8306 (60.1)
>8	2577 (32.7)	527 (8.0)	2949 (21.3)
**Birth order**			
Non previous	2486 (31.5)	2881 (43.6)	5088 (36.8)
1	3535 (44. 8)	2252 (34.1)	5557 (40.2)
2 or more	1868 (23.7)	1475 (22.3)	3187 (23.0)
**Cooking fuel**			
Clean	7848 (99.5)	6582 (99.6)	13767 (99.5)
Un clean	41 (0.5)	26 (0.4)	66 (0.5)
**Source of drinking water**			
Protected	6099 (77.3)	4052 (61.3)	10296 (74.4)
Unprotected	1788 (22.7)	2554 (38.7)	3532 (25.5)
**Toilet facility**			
Improved	5782 (72.6)	4722 (71.5)	9284 (67.1)
Unimproved	2159 (27.4)	1884 (28.5)	4545 (32.9)
**Listening to the radio**			
Not at all	4168 (52.8)	5268 (81.9)	9009 (65.1)
Yes	3719 (47.2)	1165 (18.1)	4660 (33.7)
**Watching TV**			
Not at all	238 (3.0)	447 (6.95)	659 (4.8)
Yes	7651 (97.0)	5988 (93.1)	13015 (94.1)
**Size of baby**			
Average	3871 (64.8)	3621 (66.8)	7156 (51.7)
Small	994 (16.6)	831 (15.3)	1738 (12.5)
Large	1111 (18.6)	972 (17.9)	2006 (14.5)
**Diarrhoea**			
No	6811 (86.4)	5921 (90.0)	12141 (87.8)
Yes	1069 (13.6)	658 (10.0)	1664 (12.0)
**Fever**			
No	2674 (33.9)	5503 (83.5)	7831 (56.6)
Yes	5215 (66.1)	1091 (16.5)	5993 (43.3)
**Cough**			
No	6035 (76.8)	4993 (75.7)	10494 (75.9)
Yes	1828 (23.3)	1604 (24.3)	3315 (24.0)
**Any infection**			
No	1808 (22.9)	4418 (66.9)	5942 (43.0)
Yes	6081 (77.1)	2190 (33.1)	7891 (57.0)
**Place of delivery**			
Government	391 (30.0)	5411 (98.7)	5578 (40.3)
Non-government	911 (70.0)	69 (1.3)	908 (6.6)
**Antenatal clinic visits**			
8+	427 (5.4)	487 (7.4)	869 (6.3)
4-7	3648 (46.3)	3326 (50.3)	6673 (48.2)
1-3	1572 (19.9)	1491 (22.6)	2934 (21.2)
None	2241 (28.4)	1304 (19.7)	3355 (24.3)
**Delivery assistance**			
Skilled	5861 (74.3)	5419 (82.0)	10781 (77.9)
Unskilled	2028 (25.7)	1189 (18.0)	3052 (22.1)
**Mode of delivery**			
Non-caesarean	4908 (83.0)	4010 (74.21)	8533 (61.7)
Caesarean	1002 (17.0)	1394 (25.8)	2279 (16.5)
**Postnatal checkup**			
0-2 days	428 (5.4)	753 (11.4)	1149 (8.3)
After 2 days	185 (2.4)	294 (4.5)	466 (3.4)
No	7276 (92.2)	5561 (84.2)	12217 (88.3)
**Early initiation of breastfeeding**			
After 1 hr	4916 (62.3)	4222 (63.9)	8725 (63.1)
Withing 1 hr	2973 (37.7)	2386 (36.1)	5107 (36.92)
**Ever breastfed**			
Yes	3062 (38.8)	4821 (73.0)	7587 (54.9)
No	4827 (61.2)	1787 (27.0)	6246 (45.2)
**Duration of breastfeeding**			
Up to 12 months	1674 (21.2)	2575 (39.0)	4093 (29.6)
>12 months	6216 (78.8)	4033 (61.0)	9739 (70.4)
**Literacy**			
Yes	1162 (46.7)	1169 (20.2)	2219 (16.0)
No	1328 (53.3)	4608 (79.8)	5719 (41.4)

### Data analyses

**Figure 1.**
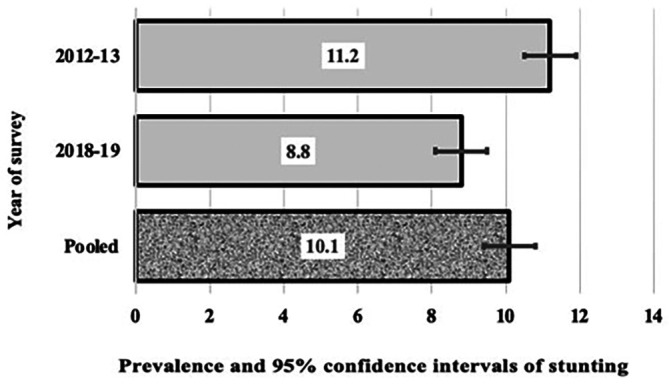
Prevalence and 95% confidence intervals (CIs) of stunting in Algerian aged children 0-23.

Descriptive analyses employed Stata ‘Svy’ (Stata Corp 17.0) commands ( https://blog.stata.com/2021/04/20/stata-17-released/) to account for the cluster sample design used in the surveys, enabling the calculation of confidence intervals for prevalence values. ^28^ Cross-tabulations were created to examine the frequencies and confidence intervals of stunting, wasting, and underweight in relation to potential confounding factors. The chi-squared test was performed to establish statistical significance. The Taylor series linearization method was used to estimate 95% confidence intervals (CIs) around the prevalence of stunting, wasting, and underweight by survey year, as shown in Figure 1. Odds ratios (ORs) were initially calculated using univariate analyses to assess the associations between stunting, wasting, and underweight. Subsequently, a stepwise backward regression model was used for multiple logistic regression analysis. Stunting, wasting, and underweight were treated as binary ependent variables. The analysis presented unadjusted ORs with 95% CIs for all potential confounding factors. Subsequently, adjusted ORs with 95% CIs were provided for variables that remained significant in the final model.

## Results

### Characteristics of the study sample

The characteristics of the study participants from the two population-based datasets (2012-13 and 2018-19) are presented in Table 1. The data for this study comprised 14498 young children aged 0-23 months in Algeria across two population-based datasets - 7890 in 2013 and 6608 in 2019. For the two population-based datasets, there was no drastic difference in the characteristics: the percentage of rural dwellers was almost equally distributed between the two population-based datasets (40% in 2012-13 vs 43 in 2018-19), and the percentage of the highest income households was similar (22% in 2012-13 and 2018-19). Other characteristics of the sample that showed little or no differences in their percentages included: boys (52% in 2012-13 and 51% in 2018-19), maternal body mass index (BMI) ≤ 18.5 kgm ^−2^ (71% in 2012-13 and 73% in 2018-19), use of clean cooking fuel (100% for each of the periods), large baby size (19% in 2012-13 and 18% in 2018-19), 8+ antenatal care (ANC) visits (5% in 2012-13 and 7% in 2018-19), and breastfeeding within one hour of delivery (38% in 2012-13 and 36% in 2018-19).

### Prevalence and 95% confidence intervals of undernutrition in 2012-13, 2018-19 and pooled

**Figure 2.**
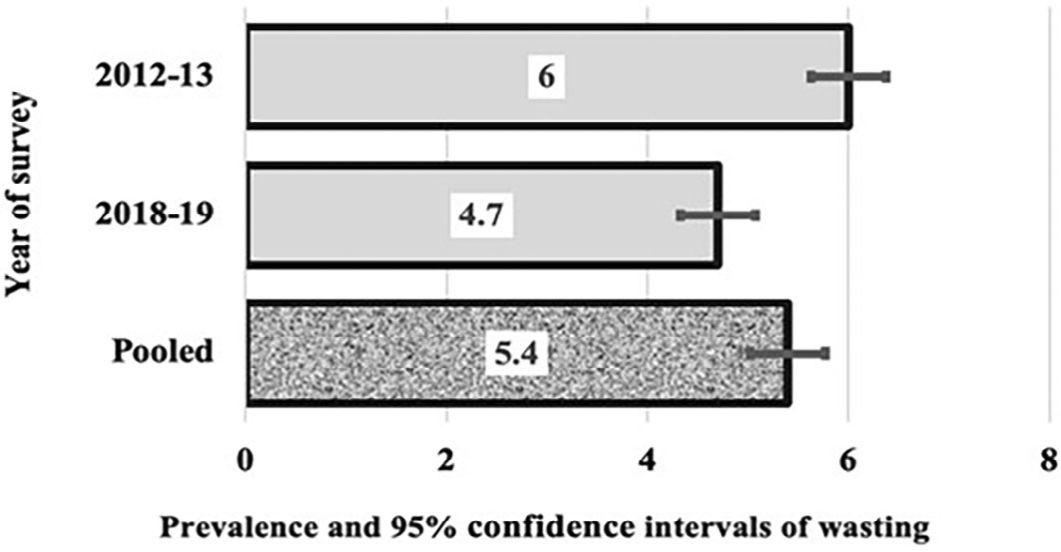
Prevalence and 95% CIs of wasting in Algerian aged children 0-23.

**Figure 3.**
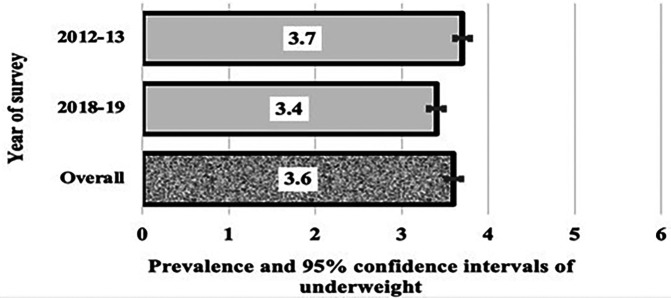
Prevalence and 95% CIs of underweight in Algerian aged children 0-23.

Figures 1, 2, and 3 show the trends in the three indices of undernutrition (stunting, wasting, and being underweight, respectively) from 2013 to 2019 in Algeria. There was a reduction in stunting in children aged under two. The prevalence of stunting significantly reduced by 2.2, i.e., the two bars did not cross each other, from 11.2% in 2013 to 8.8% in 2019 ( *p* = 0.006). In 2012-13, over one in 10 children under the age of two had stunting compared to one in twelve children under two in 2018-19. However, the overall pooled prevalence rate was 10.1%, one in ten children. The prevalence of wasting decreased from 6% to 4.7% in 2019, with a p value of 0.114% and an overall prevalence of 5.4 %. Similarly, the prevalence of underweight decreased from 3.7% to 3.4%, with a p value of 0.558, and an overall prevalence of 3.6%.

### Prevalence and unadjusted odds ratio of undernutrition

The prevalence of stunting was higher among children belonging to the poorest households [prevalence, P = 14.2%; 95% CI: (11.9, 16.7)], compared with those from the richest households [unadjusted OR: 1.61; 95% CI: (1.21, 2.13)] ( Table 2); Variables for which there was increased prevalence of stunting included being in the 18-23 months age bracket [P = 13.1%; OR: 1.40; 95% CI: (1.11, 1.75)], polygamous father [P = 9.8%; OR: 1.78; 95% CI: (1.17, 2.70)], no maternal education [P = 12.0%; OR: 1.33; 95% CI: (1.08, 1.63)], maternal BMI of 25 kg/m ^−2^ or higher [P = 45.5%; OR: 11.37; 95% CI: (6.67, 19.38)], no ANC clinics [P = 10.5%; OR: 1.45; 95% CI: (1.04, 2.01)], and breastfeeding for more than 12 months [P = 10.7%; OR: 1.28; 95% CI: (1.09, 1.51)]. Variables for which there was a decreased prevalence of stunting included large baby size at birth [P = 7.5; OR: 0.74; 95% CI: 0.57, 0.95)] and contraction of diarrhea [P = 7.8; OR: 0.73; 95% CI: 0.55, 0.99)].

**Table 2.** Prevalence and unadjusted odds ratios of undernutrition among children 0-23 months in Algeria (MICS 2012-13 &2018-19).

	Stunting			Wasting			Underweight		
Characteristics	Stunting % (95% CI)	OR (95% CI)	p-Value	Wasting % (95% CI)	OR (95% CI)	p-Value	Underweight% (95% CI)	OR (95% CI)	p-Value
**Area of residence**									
Urban	10.0 [9.1,11.1]	1.00		5.3 [4.4,6.4]	1.00		3.3 [2.8,3.8]	1.00	
Rural	10.1 [8.8,11.6]	1.01 [0.83, 1.22]	0.935	5.5 [4.4,6.8]	1.03 [0.76, 1.39]	0.864	4.0 [3.1,5.1]	1.22 [0.89, 1.67]	0.221
**Wealth Index**									
Richest	9.3 [7.8,11.2]	1.00		5.9 [4.0,8.8]	1.00		3.3 [2.4,4.5]	1.00	
Fourth	9.0 [7.5,10.8]	0.97 [0.73, 1.29]	0.809	5.1 [3.8,6.7]	0.85 [0.51, 1.42]	0.535	2.9 [1.9,4.2]	0.86 [0.53,1.39]	0.53
Middle	9.2 [7.8,10.9]	0.99 [0.75, 1.30]	0.935	4.8 [3.6,6.2]	0.79 [0.47, 1.33]	0.378	3.4 [2.5,4.4]	1.01 [0.66, 1.55]	0.953
Poorer	9.1 [7.6,10.8]	0.97 [0.74, 1.28]	0.845	5.2 [4.1,6.7]	0.88 [0.54, 1.43]	0.601	3.9 [2.9,5.2]	1.17 [0.77, 1.81]	0.459
Poorest	14.2[11.9,16.7] ***	1.61 [1.21, 2.13]	0.001	6.0 [4.4,8.0]	1.01 [0.59, 1.71]	0.984	4.6 [3.4,6.2]	1.40 [0.90, 2.18]	0.135
**Sex of baby**									
Boy	12.2[11.1,13.5]	1.00		6.1 [4.9,7.4]	1.00		4.2 [3.5,5.1]	1.00	
Girl	7.7 [6.8,8.7] ***	0.60 [0.51, 0.71]	<0.001	4.7 [3.9,5.6]	0.76 [0.57, 1.01]	0.061	2.9 [2.3,3.6] **	0.67 [0.50, 0.90]	0.008
**Child age**									
0-5	9.7 [8.3,11.4]	1.00		10.5[8.9,12.3]	1.00		7.3 [6.0,8.8]	1.00	
6-11	7.7 [6.5,9.0]	0.77 [0.60, 0.99]	0.038	5.7 [3.9,8.2]	0.51 [0.33, 0.80]	0.003	3.8 [2.8,5.0]	0.50 [0.34, 0.72]	<0.001
12-17	9.9 [8.5,11.5]	1.02 [0.81, 1.29]	0.865	3.3 [2.4,4.5]	0.29 [0.21, 0.41]	<0.001	1.2 [0.8,1.8]	0.16 [0.10, 0.25]	<0.001
18-23	13.1[11.4,14.9] ***	1.40 [1.11, 1.75]	0.004	2.1 [1.4,2.9] ***	0.18 [0.12, 0.27]	<0.001	2.0 [1.4,2.9] ***	0.26 [0.17, 0.39]	<0.001
**Mother's age**									
15-19 years	10.6 [9.1,14.8]	1.00		7.0 [4.8,10.2]	1.00		5.2 [3.3,8.1]	1.00	
20-34	9.7 [8.8,10.7]	0.82 [0.62, 1.08]	0.156	5.2 [4.5,6.0]	0.73 [0.48, 1.09]	0.12	3.6 [3.1,4.3]	0.68 [0.43,1.10]	0.119
35-49 years	10.6 [9.4,11.9]	0.90 [0.69, 1.18]	0.441	4.9 [4.1,5.9]	0.68 [0.45, 1.03]	0.069	3.2 [2.6,4.0]	0.60 [0.37, 0.99]	0.047
**Father's age**									
18-34	9.5 [8.0,11.3]	1.00		7.4 [5.8,9.2]	1.00		4.1 [3.1,5.5]	1.00	
35-44	10.0 [9.0,11.1]	1.06 [0.85, 1.32]	0.601	4.6 [4.0,5.4]	0.61 [0.47, 0.80]	<0.001	3.5 [2.9,4.1]	0.84 [0.59, 1.18]	0.304
45+	10.3 [8.8,12.1]	1.09 [0.85, 1.41]	0.485	4.4 [3.5,5.5] ***	0.58 [0.41, 0.80]	0.001	2.7 [2.1,3.6]	0.66 [0.45, -.96]	0.03
**Mother's age at marriage**									
≤18 years	11.2 [9.0,14.0]	1.00		6.7 [4.8,9.3]	1.00		4,6 [3.2,6.5]	1.00	
>18 years	9.9 [9.1,10.8]	0.87 [0.67, 1.13]	0.296	4.9 [4.4,5.6]	0.72 [0.50, 1.06]	0.094	3.4 [2.9,3.9]	0.73 [0.50, 1.07]	0.108
**Marital status**									
Married	10.0 [9.2,10.8]	1		5.1 [4.5,5.7]	1		3.5 [3.0,4.0]		
Not married	10.8 [9.0,13.0]	1.10 [0.89, 1.34]	0.376	6.0 [4.4,8.0]	1.18 [0.88, 1.60]	0.272	4.4 [3.1,6.2]	1.28 [0.90, 1.84]	0.172
**Polygymstatus**									
No	16.3 [9.0,10.7]	1.00		5.1 [4.5,5.8]	1.00		3.5 [3.0,4.0]	1.00	
Yes	9.8 [11.4,22.7] ***	1.78 [1.17, 2.70]	0.007	3.5 [1.8,6.5]	0.67 [0.34, 1.31]	0.239	3.6 [1.9,7.0]	1.05 [0.52, 2.13]	0.882
**Maternal education**									
Secondary and above	9.3 [8.2,10.6]	1.00		4.9 [4.1,5.9]	1.00		3.1 [2.5,3.8]	1.00	
Primary	10.0 [8.8,11.3]	1.08 [0.90, 1.30]	0.389	5.7 [4.8,6.7]	1.17 [0.92, 1.48]	0.212	4.2 [3.4,5.1]	1.36 [1.05, 1.77]	0.021
No education	12 [10.4,13.7] *	1.33 [1.08, 1.63]	0.007	5.0 [4.1,6.2]	1.03 [0.78, 1.36]	0.852	3.6 [2.9,4.5]	1.16 [0.86, 1.58]	0.327
**Father education**									
Secondary and above	9.1 [7.9,10.4]	1.00		5.4 [4.1,7.1]	1.00		2.8 [2.1,3.6]	1.00	
Primary	9.9 [8.7,11.2]	1.10 [0.91, 1.35]	0.342	5.3 [4.4,6.3]	0.97 [0.69, 1.37]	0.876	4.3 [3.5,5.3]	1.57 [0.12, 2.23]	0.011
No education	12.1[10.6,14.1] **	1.39 [1.11, 1.75]	0.004	5.6 [4.4,7.0]	1.03 [0.71, 1.49]	0.866	3.6 [2.8,4.5] *	1.29 [0.90, 1.85]	0.160
**Maternal BMI**									
≤18.5	6.8 [6.1,7.7]	1.00		-	-		4.8 [4.1,5.5]	1.00	
19-25	17.6[15.7,19.6]	2.91 [2.46, 3.43]	<0.001	-	-		0.3 [0.1,0.5]	0.52 [0.03, 0.11]	<0.001
25+	45.5[33.3,58.2] ***	11.37 [6.67, 19.38]	<0.001	-	-		0.7 [0.1,5.1] ***	0.15 [0.02, 1.08]	0.059
**Household members**									
1-3	8.6 [7.2,10.2]	1.00		4.7 [3.7,5.9]	1.00		4.0 [3.1,5.3]	1.00	
4-8	10.4 [9.4,11.4]	1.23 [1.00, 1.52]	0.049	4.8 [4.2,5.5]	1.04 [0.78, 1.38]	0.803	3.0 [2.5,3.5]	0.73 [0.52, 1.02]	0.064
>8	10.5 [8.8,12.5]	1.25 [0.95, 1.65]	0.106	7.6 [5.2,11.0] *	1.68 [1.03, 2.73]	0.036	4.8 [3.5,6.7] *	1.20 [0.76, 1.88]	0.436
**Birth order**									
Non previous	9.2 [7.9,10.6]	1.00		5.5 [4.5,6.8]	1.00		4.2 [3.3,5.2]	1.00	
1	10.8 [9.6,12.1]	1.20 [0.98, 1.46]	0.075	4.9 [4.2,5.9]	0.89 [0.67, 1.19]	0.432	3.2 [2.5,4.1]	0.75 [0.53, 1.07]	0.112
2 or more	10.2 [8.9,11.8]	1.13 [0.91, 1.40]	0.254	6.0 [4.1,8.7]	1.09 [0.69, 1.71]	0.721	3.3 [2.7,4.2]	0.80 [0.57, 1.11]	0.178
**Cooking fuel**									
Clean	10.1[9.310.9]	1.00		5.4 [4.7, 6.2]	1.00		3.6 [3.1, 4.1]	1.00	
Un clean	7.5 [3.415.6]	0.72 [0.32, 1.66]	0.445	2.4 [0.8, 6.9]	0.43 [0.14, 1.31]	0.137	3.0 [0.9, 9.6]	0.85 [0.25,2.87]	0.789
**Source of drinking water**									
Protected	10.0 [9.1,11.0]	1.00		5.7 [4.8,6.7]	1.00		3.8 [3.2,4.5]	1.00	
Unprotected	10.1 [8.6,11.8]	1.00 [0.83, 1.22]	0.962	4.6 [3.7,5.7]	0.80 [0.60, 1.08]	0.151	2.9 [2.2,3.8]	0.74 [0.53, 1.04]	0.087
**Toilet facility**									
Improved	10 [9.1, 11.0]	1.00		6.0 [5.0, 7.1]	1.00		4.0 [3.4, 4.7]	1.00	
Unimproved	10.2 [8.8, 11.7]	1.02 [0.85, 1.23]	0.833	4.2 [3.4, 5.2] **	0.69 [0.52, 0.93]	0.013	2.7 [2.0, 3.5] **	0.66 [0.47, 0.91]	0.013
**Listening to the radio**									
Not at all	9.7 [8.7,10.7]	1.00		4.9 [4.2,5.8]	1.00		3.6 [3.0,4.3]	1.00	
Yes	11.1 [9.8,12.5]	1.16 [0.98, 1.39]	0.090	5.9 [4.8,7.2]	1.20 [0.92, 1.57]	0.187	3.5 [2.7,4.5]	0.97 [0.71, 1.33]	0.870
**Watching TV**									
Not at all	12.1 [8.9,16.3]	1.00		7.0 [4.7,10.3]	1.00		4.0 [2.5,6.6]	1.00	
Yes	1.0 [9.2,10.9]	0.81 [0.57, 1.15]	0.245	5.2 [4.6,5.9]	0.72 [0.47, 1.12]	0.147	3.6 [3.1,4.1]	0.88 [0.52, 1.49]	0.633
**Size of baby**									
Average	9.9 [8.9,10.9]	1.00		4.9 [4.3,5.6]	1.00		3.1 [2.6,3.7]	1.00	
Small	14.0[12.0,16.3]	1.49 [1.22, 1.82]	<0.001	7.1 [5.5,9.2]	1.49 [1.10, 2.01]	0.010	7.0 [5.5,8.9]	2.37 [1.75, 3.22]	<0.001
Large	7.5 [6.0,9.2] ***	0.74 [0.57, 0.95]	0.018	3.6 [2.8,4.8] ***	0.73 [0.54, 0.99]	0.045	1.7 [1.2,2.3] ***	0.53 [0.36, 0.78]	0.001
**Diarrhoea last two weeks**									
No	10.3 [9.5,11.3]	1.00		5.1 [4.5,5.8]	1.00		3.4 [2.9,4.0]	1.00	
Yes	7.8 [6.0,10.1] *	0.73 [0.55, 0.99]	0.040	7.6 [4.4,13.0]	1.54 [0.84, 2.83]	0.160	4.6 [3.0,7.0]	1.37 [0.85, 2.19]	0.193
**Fever**									
No	9.4 [8.4,10.5]	1.00		5.5 [4.4,6.7]	1.00		3.8 [3.2,4.6]	1.00	
Yes	10.9 [9.9,12.1] *	1.18 [1.01, 1.38]	0.034	5.3 [4.6,6.2]	0.97 [0.76, 1.25]	0.839	3.3 [2.7,3.9]	0.85 [0.67, 1.07]	0.154
**Cough**									
No	9.9 [9.0,10.9]	1.00		5.3 [4.7,6.0]	1.00		3.7 [3.2,4.3]	1.00	
Yes	10.5 [9.0,12.3]	1.07 [0.88, 1.31]	0.482	5.7 [3.7,8.7]	1.09 [0.68, 1.75]	0.722	3.0 [2.1,4.5]	0.80 [0.53, 1.23]	0.324
**Any infection**									
No	9.5 [8.3,10.8]	1.00		5.2 [4.4,6.2]	1.00		3.7 [3.0,4.5]	1.00	
Yes	10.5 [9.5,11.5]	1.12 [0.95, 1.32]	0.162	5.5 [4.5,6.8]	1.07 [0.81, 1.41]	0.652	3.5 [2.9,4.3]	0.96 [0.72,1.28]	0.780
**Place of delivery**									
Government	9.4 [8.2,10.7]	1.00		4.6 [3.9,5.4]	1.00		3.6 [3.0,4.4]	1.00	
Non-government	9.7 [7.4,12.6]	1.04 [0.75, 1.44]	0.824	4.9 [3.3,7.4]	2.07 [0.68, 1.70]	0.760	2.9 [1.7,4.8]	0.79 [0.45, 1.40]	0.428
**Antenatal clinic visits**									
8+	7.5[5.6,9.9]	1.00		3.8 [2.4,5.8]	1.00		3.9 [2.6,5.9]	1.00	
4-7	9.6 [8.6,10.7]	1.31 [0.95, 1.82]	0.103	5.0 [4.3,5.9]	1.35 [0.82, 2.21]	0.236	2.9 [2.4,3.5]	0.74 [0.46, 1.19]	0.216
1-3	11.5[10.1,13.0]	1.61 [1.14, 2.26]	0.006	5.3 [4.4,6.4]	1.44 [0.89, 2.34]	0.141	4.5 [3.6,5.5]	1.16 [0.72, 1.88]	0.544
None	10.5 [9.0,12.1] *	1.45 [1.04, 2.01]	0.029	6.6 [4.9,8.9]	1.81 [1.05, 3.10]	0.032	4.0 [3.0,5.3] *	1.04 [0.62, 1.73]	0.889
**Delivery assistance**									
Skilled	10.0 [9.2,10.9]	1.00		5.0 [4.5,5.6]	1.00		3.4 [3.0,3.9]	1.00	
Unskilled	10.2 [8.7,11.9]	1.01 [0.85, 1.21]	0.875	6.7 [4.8,9.2]	1.35 [0.98, 1.87]	0.066	4.1 [3.0,5.5]	1.20 [0.90, 1.62]	0.218
**Mode of delivery**									
Non-caesarean	9.8 [8.5,11.3]	1.00		4.4 [3.6,5.3]	1.00		3.3 [2.7,4.2]	1.00	
Caesarean	10.4 [9.5,11.5]	1.07 [0.89, 1.28]	0.459	5.5 [4.8,6.3]	1.27 [0.99, 1.62]	0.058	3.5 [2.9,4.1]	1.04 [0.78, 1.40]	0.773
**Postnatal check-up **									
0-2 days	8.9 [6.7,11.6]	1.00		6.2 [4.5,8.5]	1.00		3.6 [2.6,5.1]	1.00	
After 2 days	10.1 [7.0,14.3]	1.15 [0.73, 1.83]	0.549	5.6 [3.6,8.7]	0.90 [0.51, 1.61]	0.729	4.1 [2.4,7.1]	1.15 [0.59, 2.22]	0.678
No	10.2[9.3,11.1]	1.17 [0.86, 1.58]	0.324	5.3 [4.5,6.2]	0.85 [0.58, 1.23]	0.383	3.5 [3.0,4.1]	0.98 [0.68, 1.42]	0.920
**Early initiation of breastfeeding**									
After 1 hr	10.0 [9.1,11.1]	1.00		5.5 [4.6,6.5]	1.00		4.1 [3.4,4.8]	1.00	
Withing 1 hr	10.1 [9.0,11.3]	1.01 [0.87, 1.17]	0.937	5.3 [4.4,6.2]	0.96 [0.78, 1.18]	0.705	2.7 [2.2,3.3] ***	0.66 [0.51, 0.84]	0.001
**Ever breastfed**									
Yes	9.9 [8.9,11.0]	1.00		4.8 [4.1,5.5]	1.00		3.3 [2.8,3.8]	1.00	
No	10.3 [9.2,11.5]	1.04 [0.89, 1.22]	0.620	6.2 [5.0,7.6] *	1.32 [1.05, 1.66]	0.018	3.9 [3.2,4.9]	1.21 [0.95, 1.55]	0.119
**Duration of breastfeeding**									
Up to 12 months	8.5 [7.4,9.8]	1.00		6.9 [5.8,8.1]	1.00		4.9 [4.1,5.8]	1.00	
>12 months	10.7 [9.8,11.7] **	1.28 [1.09, 1.51]	0.003	4.8 [4.0,5.7] ***	0.68 [0.54, 0.84]	<0.001	3.0 [2.5,3.7] ***	0.61 [0.48, 0.77]	<0.001
**Literacy**									
Yes	10.0 [8.4,11.8]	1.00		5.4 [4.3,6.8]	1.00		4.4 [3.4,5.6]	1.00	
No	9.3 [8.2,10.5]	0.93 [074, 1.15]	0.489	5.0 [4.2,6.0]	0.92 [0.69, 1.21]	0.539	3.3 [2.7,4.0]	0.75 [0.55, 1.01]	0.061

- Omitted values.

Confidence intervals (CI) =

* P< 0.05 .

** p<0.01 .

*** p<0.001.

Variables for which there was an increased prevalence of wasting included belonging to a household with more than eight members [P = 7.6%; OR: 1.68; 95% CI: 1.03, 2.73)] and not having ever been breastfed [P = 6.2%; OR: 1.32; 95% CI: 1.05, 1.66)]. Variables for which there was a decreased prevalence of wasting included belonging to the 18-23 months age group [P = 2.1; OR: 0.18; 95% CI: (0.12, 0.27)], paternal age > 45 years [P = 4.4%; OR: 0.58; 95% CI: (0.41, 0.80)], large size at birth [P = 3.6%; OR: 0.73; 95% CI: (0.54, 0.99)], and being breastfed for more than 12 months [P = 4.8%; OR: 0.68; 95% CI: (0.54, 0.84)].

Variables for which there was an increased prevalence of underweight included no ANC clinic visits [P = 4.0%; OR: 1.04; 95% CI: 0.62, 1.73)] and no maternal education [P = 3.6%; OR: 1.29; 95% CI: 0.90, 1.85)]. Variables for which there was an increased prevalence of being underweight included being put to the breast within one hour after birth [P = 2.7%; OR: 0.66; 95% CI: (0.51, 0.84)], having been breastfed for more than 12 months [P = 3.0%; OR: 0.61; 95% CI: (0.48, 0.77)], being a girl [P = 2.9%; OR: 0.67; 95% CI: (0.50, 0.70)], belonging to the 18-23 months age group [P = 2.0%; OR: 0.26; 95% CI: (0.17, 0.39)], maternal BMI of 25 kg/m ^−2^ or higher [P = 0.7%; OR: 0.15; 95% CI: (0.02, 1.08)], and being of a large size at birth [P = 1.7%; OR: 0.53; 95% CI: (0.36, 0.78)].

### Factors associated with undernutrition

The significant factors associated with the three undernutrition categories (stunting, wasting, and underweight) are presented in Table 3. Significantly decreased odds of stunting occurred in the period 2018-19 [adjusted odds ratio (AOR) = 0.80; 95% CI: 0.66, 0.97]. The odds of stunting were significantly higher among children from the poorest households compared with those from the richest households [AOR = 1.56; 95% CI: (1.16, 2.10)]. The odds of being stunted were significantly higher among children aged 18-23 months [AOR = 1.36; 95% CI): (1.10, 1.69)]. Children whose mothers had a BMI of 25 kg/m ^2^ or over were significantly more likely to be stunted than their counterparts whose mothers had a BMI less than 18.5 kg/m ^2^ [AOR = 14.38; 95% CI: (7.86, 26.30)]. The odds of being stunted were significantly higher among children who were perceived as small at birth than among those who were perceived as average-sized (AOR = 1.72; 95% CI: (1.39, 2.12)]. Children whose mothers attended between 1 and 3 ANC clinics were significantly more likely to be stunted than those who attended 8 or more ANC clinics [AOR = 1.63; 95% CI: (1.15, 2.33)]. In contrast, decreased odds of stunting were associated with being female [AOR = 0.59; 95% CI: 0.50, 0.70)].

**Table 3.** Adjusted odds ratios for determinants of undernutrition among children 0-23 months in Algeria (MICS 2012-13 &2018-19).

	Stunting		Wasting		Underweight	
Characteristics	A OR (95%)	p-Value	A OR (95%)	p-Value	A OR (95%)	p-Value
**Year**						
2012-13	1.00		1.00		1.00	
2018-19	0.80 [0.66, 0.97]	0.024	0.87 [0.65, 1.15]	0.319	1.11 [0.83, 1.49]	0.488
**Wealth Index**						
Richest	1.00					
Fourth	0.89 [0.67, 1.17]	0.392				
Middle	0.92 [0.70, 1.21]	0.562				
Poorer	0.94 [0.71, 1.24]	0.663				
Poorest	1.56 [1.16, 2.10]	0.003				
**Sex of baby**						
Boy	1.00				1.00	
Girl	0.59 [0.50, 0.70]	<0.001			0.67 [0.51, 0.87]	0.003
**Child age (months)**						
0-5	1.00		1.00		1.00	
6-11	0.56 [0.44, 0.72]	<0.001	0.47 [0.34, 0.64]	<0.001	0.60 [0.42, 0.85]	0.004
12-17	0.87 [0.69, 1.09]	0.230	0.30 [0.22, 0.41]	<0.001	0.20 [0.14, 0.30]	<0.001
18-23	1.36 [1.10, 1.69]	0.005	0.17 [0.12, 0.24]	<0.001	0.27 [0.18, 0.41]	<0.001
**Father's age**						
18-34			1.00			
35-44			0.73 [0.55, 0.98]	0.039		
45+			0.72 [0.51, 1.01]	0.060		
**Maternal education**						
Secondary and above					1.00	
Primary					1.47 [1.05, 2.06]	0.023
No education					1.33 [0.95, 1.86]	0.098
**Maternal BMI**						
≤18.5	1.00				1.00	
19-25	3.44 [2.91, 4.07]	<0.001			0.07 [0.03, 0.16]	<0.001
25+	14.38 [7.86,26.30]	<0.001			0.20 [0.03, 1.53]	0.122
**Size of baby**						
Average	1.00		1.00		1.00	
Small	1.72 [1.39, 2.12]	<0.001	1.50 [1.11, 2.03]	0.009	2.10 [1.54, 2.85]	<0.001
Large	0.65 [0.51, 0.83]	0.001	0.78 [0.58, 1.07]	0.120	0.55 [0.37, 0.82]	0.004
**Antenatal clinic visits**						
8+	1.00		1.00			
4-7	1.31 [0.94, 1.82]	0.115	1.39 [0.84, 2.29]	0.204		
1-3	1.63 [1.15, 2.33]	0.007	1.60 [0.97, 2.63]	0.064		
None	1.77 [1.14, 2.75]	0.011	1.82 [1.00, 3.29]	0.048		
**Early initiation of breast feeding**						
After 1 hr					1.00	
Within 1 hr					0.67 [0.51, 0.87]	0.003

Increased odds of wasting were significantly associated with being perceived to be small at birth [AOR = 1.50; 95% CI: 1.11, 2.03)] and being a child to a mother who attended no ANC clinics [AOR = 1.82; 95% CI: (1.00, 3.29)]. Decreased odds of wasting were associated with being aged 18-23 months [AOR = 0.17; 95% CI: 0.12, 0.24)] and having a father aged 35-44 years [AOR = 0.73; 95% CI: (0.55, 0.98)].

The likelihood of being underweight was significantly higher among children whose mothers had primary education only than among their counterparts whose mothers had secondary school education or higher [AOR = 1.47; 95% CI: 1.05, 2.06)]. Children who were perceived to be small at birth were significantly more likely to be underweight than those who were perceived to be average-sized [AOR = 2.10; 95% CI: (1.54, 2.85)]. The odds of being underweight were significantly lower among girls than among boys [AOR = 0.67; 95% CI: (0.51, 0.87)]. Children aged 12-17 months were significantly less likely to be underweight than those aged 0-5 months [AOR = 0.20; 95% CI: (0.14, 0.30)]. Children whose mothers had a BMI of 19-25 kg/m ^2^ were less likely to be underweight than those whose mothers had a BMI of 18.5 kg/m ^2^ or less [AOR = 0.17; 95% CI: 0.12, 0.24)]. Underweight odds were significantly lower among children who were to the breast within 1 h after birth compared with those who were to the breast after 1 h (AOR = 0.67; 95% CI: [0.51, 0.87]).

## Discussion

The current study aimed to investigate the prevalence of the three indicators of undernutrition among Algerian children under two years of age and to identify the significant factors associated with undernutrition to make progress in combating childhood undernutrition by 2030. This study combined data from the Algerian MICS surveys conducted in 2012-13 and 2018-19. Our analysis revealed a significant decline in the prevalence of stunting in the two population-based datasets. The prevalence of undernutrition was generally lower in the 2018-19 dataset compared than in the 2012-13 dataset. Multivariate analysis identified several key factors associated with undernutrition. Notably, the factors that were significant determinants across all three indices of undernutrition were child’s age and perceived size at birth. ANC visits were strongly associated with stunting and wasting, while maternal BMI and male sex were significant factors affecting both stunting and being underweight. Furthermore, we observed associations between household wealth index and the year of the survey with stunting, as well as between advanced paternal age and wasting. Maternal education level and the timing of breastfeeding initiation were also notable factors specifically associated with being underweight.

About one in ten children in Algeria under the age of two in Algeria experienced stunting, with a positive improvement between 2012 and 2019. Contrary to the study findings, the number of children under five years of age with undernutrition worldwide has been increasing since 2014. A recent FAO report indicated that the overall prevalence of undernutrition in Africa increased from 19.4% in 2021 to 19.7% in 2022. In contrast, the prevalence of undernutrition in Asia declined from 8.8% in 2021 to 8.5% in 2022, representing a reduction of over 12 million individuals. ^29^ It should be emphasized that the last nationally accessible survey for Algeria dates back to 2019, and no further data have been gathered or published since then. Owing to the lack of recent nationally representative data after 2019, it is difficult to determine whether the current undernutrition in Algeria after 2019 is improving or worsening, highlighting the importance of consistent data collection. These data are crucial for combating undernutrition, a complex public health issue influenced by multiple interconnected factors. ^15^ These factors are vulnerable to various crises, both nationally and globally, such as pandemics, environmental catastrophes, civil unrest, and global conflicts. ^11– 13^ Consistent data collection can help to better understand and manage undernutrition, leading to more effective interventions and policymaking. ^30^

The study analyses demonstrated that the prevalence of stunting was significantly higher among children belonging to the poorest households, consistent with previous research that identified an association between disparity in socioeconomic status and child health outcomes, particularly stunting. ^31, 32^ Inequality in stunting is associated with variations in access to healthcare, inadequate nutrition, and exposure to environmental contaminants, including pathogens from inadequate water, sanitation, and living conditions. ^33^ Furthermore, there is evidence that significant gaps exist between the wealthiest and poorest 20% of the population across all age groups. In the aggregated sample, children from the lowest income families exhibited a peak stunting prevalence of 50% at 29 months, but the prevalence among those with the greatest wealth was significantly lower, 22% at 27 months. Moreover, wealthy countries seem to achieve maximum stunting at relatively early ages. ^34^

In the current study, stunting was strongly correlated with age in children aged 18-23 months. This finding is consistent with past research, which observed that the prevalence of stunting generally remained high among children aged 2–4 years. Recent studies in low- and middle-income countries concur with the finding that the prevalence of stunting increases with a child’s age. ^15, 35, 36^ This observation can be attributed to earlier deprivation, as stunting results from the accumulation of adverse exposures. The study findings may be attributed to the protective effect of breastfeeding, which is buttressed by the Islamic religion, which recommends that mothers breastfeed their babies for two years; mothers often aim to breastfeed their babies until the age of two years. ^37^ However, despite Algeria being a Muslim country, exclusive breastfeeding in the first six months is still low (about 28.6%), even though it is still better than the rate in Egypt (13%), ^38^ Tunisia (13.5%) ^39^ and Libya (25.2%). ^40^ The increased odds of stunting observed in older children may be attributed to inappropriate complementary feeding during the weaning period. ^41^ Complementary feeding practices in the Middle East and North Africa do not meet the recommendations, with < 50% of children meeting their minimum dietary diversity (MDD) in the five target countries. ^42^

The current study observed increased odds of stunting and being underweight among mothers with limited education. Similar findings were found in Pakistan, in a sample of 3071 Pakistani children aged 0-59 months from the Pakistan Demographic and Health Surveys (DHS) 2012-2013 found a significant association between undernutrition among children under five and mothers with low education levels. ^43^ Similarly, a study conducted in Ethiopia aimed to evaluate stunting and its related factors in children aged 6 59 months. ^44^ A systematic study and meta-analysis conducted in middle-income countries also found a statistically significant inverse correlation between maternal educational level and probability of being underweight. ^45^ This finding can be explained by the ability of educated mothers to have a more comprehensive understanding of the health issues related to their children. Parents without formal education encounter challenges in interacting with healthcare providers, expressing their children’s symptoms of illness, and understanding information related to their children’s health. ^46^ Inequality puts considerable pressure on family financial resources, restricts the utilization of healthcare services, and impedes accessibility to nutritious foods. These conditions adversely affect the nutritional health of mothers and children, increasing their susceptibility to diseases and growth disorders. ^37^

A recent study indicated that boys are considerably more prone to stunting than are girls. This disparity may be linked to behavioral variations, disparities between sexes, and the genetic susceptibility of boys to illness during early childhood. ^47^ Similarly, a meta-analysis focusing on children under five years of age in Sub-Saharan Africa found that boys in sub-Saharan Africa had higher odds of being stunted in their early childhood than girls. ^48^ Furthermore, previous research has suggested that boys tend to grow slightly faster than girls. ^49^ However, they are vulnerable to nutritional deficiencies and infections. However, a meta-analysis of DHS data from 16 sub-Saharan countries ^48^ revealed that sex-based differences in feeding practices within families might contribute to a higher risk of stunting among boys. In many African cultures, girls are valued more than boys because of their potential to contribute to agricultural labor and their perceived worth in terms of future dowry payments. Consequently, girls tend to receive better nutrition and care in many low-income African settings, which may explain why boys are more likely to experience stunting. ^50^ However, according to a meta-analysis, undernutrition is still a consequence of an insufficient intake of nutritious food. Moreover, a previous study showed that in households experiencing food insecurity, there is a greater probability of undernutrition in girls than in boys. ^51^

The findings indicated a significant association between perceived small birth size and a higher likelihood of wasting in children. This finding is consistent with those of a previous study conducted in Nigeria. ^52^ Other studies from Ethiopia, ^53^ Brazil ^54^ and Pakistan ^55^ corroborated this finding as they observed that perceived baby size was an accurate predictor of child growth. However, it is important to approach this finding cautiously as the methodology mothers use to estimate their babies’ sizes is not clearly understood. Insufficient maternal nutrition during pregnancy may result in low birth weight, because the developing fetus depends solely on the mother’s nutrient provision through the placenta. As a result, any nutritional deficiencies experienced by the mother can impede the fetus’s growth and development, which underscores the critical importance of prioritizing women’s health and prenatal care, ^56^ thereby empowering mothers to give their children a stronger foundation for life. Children of mothers who did not visit any ANC clinic were significantly more prone to wasting. Previous research demonstrated a significant correlation between mothers’ lack of knowledge of child feeding practices and the occurrence of wasting. ^57^ This corroborates our finding of an association between ANC clinic attendance and wasting, as it is at such places that mothers gain the requisite knowledge of appropriate child-feeding practices, which may be applied to prevent wasting. Children aged 18-23 months were less likely to be wasted, consistent with the findings of a previous study in Bangladesh, which indicated that the odds of wasting decreased as the child’s age increased, ^58^ which was corroborated by previous studies. ^59, 60^

This study found that maternal BMI was inversely related to child being underweight. The mother’s BMI has been found to affect the nutritional status of a child during the pre- and post-pregnancy periods. ^51^ Undernourishment in women of reproductive age, during pregnancy, and in children within the first two years of life creates a substantial health burden, underscoring the need for focused treatments for these groups. ^61^ Furthermore, undernourished mothers frequently face challenges that hinder their ability to breastfeed and reduce their mental and physical vitality. This could adversely affect her capacity to care for her child. ^62^ A previous study conducted in Rwanda documented an association between inadequate maternal nutrition and poor nutritional results. ^63^ In Ghana, another study revealed that a mother’s BMI negatively correlated with child being underweight. ^64^

The present study found that older children aged 18-23 months were less prone to underweight. The results of this study are consistent with the findings of Eriga et al. and Pheringxay et al., which found that children aged 13-24 months and 1212–24 months have a greater chance of being underweight. ^65, 66^ The positive association between 12-23 months and being underweight could be attributed to the fact that mothers usually pay less attention to their children when they get older, which could be because of attending work. At this stage, older siblings, other family members, or childcare centers assume the role of caregivers, which may lead to inappropriate feeding and hygiene practices. ^67^ This ultimately makes these children more susceptible to childhood illnesses, resulting in underweight. ^67^ According to the study analysis, breastfeeding within the first hour after birth was inversely associated with being underweight. Similarly, a previous study in Ghana reported an increased risk of wasting among children who were not breastfed within the first hour of birth. ^68^ This is corroborated by previous studies. ^69– 71^ A study by Congo emphasized the correlation between early breastfeeding and child undernutrition, underscoring that early breastfeeding provides numerous advantages, particularly the delivery of colostrum, the initial milk produced by a new mother, which is rich in protective properties. ^69^

Improving nutrition in Algeria, particularly for women and children, is crucial for child survival, and plays a key role in the country’s economic development. Allocating resources toward nutrition is a cost-effective strategy for preventing the heavy burden of malnutrition. Additionally, investing in nutrition positively affects the health, education, and agricultural sectors. ^72, 73^ Economic approaches to addressing malnutrition can significantly boost a nation’s economy. Nutrition programs built on effective community engagement can deliver considerable social and economic benefits such as improved quality of life, increased productivity, and strong economic returns. ^72^ For children, these investments enhance cognitive abilities, school attendance, and academic performance, resulting in long-term benefits for the educational sector. ^74^ Given these advantages, Algeria should establish comprehensive national nutrition policies. Although the country currently has legislation and specific policies addressing undernutrition, these do not fully address the rising issue of overnutrition. ^75^ A well-rounded nutrition policy is necessary to foster sustainable economic growth and development, targeting both undernutrition and overnutrition to ensure optimal nutrition for all and to prevent malnutrition in all forms.

To address the pressing issue of child undernutrition in Algeria, it is recommended that the implementation of a multifaceted national nutrition policy prioritize community-based interventions to improve access to nutritious food, clean water, and healthcare services, particularly in rural and disadvantaged areas, support for poverty reduction initiatives and social protection programs to address economic inequality and food insecurity, and collaboration with international organizations and stakeholders to leverage resources, expertise, and best practices in addressing child undernutrition. By adopting this comprehensive approach, Algeria can make significant strides in reducing child undernutrition, promoting healthy growth and development and fostering a strong foundation for sustainable economic growth and development.

One of the strengths of this study was that it utilized a large sample by pooling two different population-based datasets to examine changes in the prevalence of undernutrition and related determinants among Algerian children under the age of two years, enhancing statistical power and the ability to compare outcomes, whereas findings from a single study could be inconclusive. Another strength of our study is its exclusive focus on undernutrition in Algeria, which allowed for an in-depth analysis of the contributing factors specific to this country. By narrowing our focus, we gained detailed insight into the changes, unique challenges, and circumstances influencing undernutrition in this geographic area. Additionally, our study used the most recent nationally representative data from two periods with high responsive rates (97-99%), ensuring that our findings reflect the entire population and enhancing their generalizability and applicability. The implementation of a population-based design and a substantial pooled sample size guarantee that the obtained data accurately represent the entire population within the target area. This method improved the validity and reliability of results by reducing selection bias and enabling more accurate estimations of prevalence and relationships.

Nonetheless, this study had several limitations. MICS surveys are predominantly cross-sectional and collect data at a single moment, thereby reducing the capacity to ascertain causal associations or comprehend the dynamic causes of undernutrition. Moreover, cross-sectional statistics depend on self-reported information, which may be susceptible to recall or social desire bias, potentially resulting in errors. Finally, unaccounted for confounding variables may have affected the study outcomes.

## Conclusion

Child undernutrition remains a persistent and complex challenge in Algeria, with far-reaching consequences for individual health, economic productivity, and national development. The findings of this study underscore the need for a comprehensive and multifaceted approach to address the root causes of undernutrition, including poverty, limited access to healthcare and education, and inadequate nutritional knowledge. By prioritizing nutrition-sensitive interventions, investing in human capital, and fostering a coordinated response across sectors, Algeria can break the cycle of undernutrition and unlock a brighter future for its children. Ultimately, ensuring the optimal nutrition and health of Algerian children is not only a moral imperative, but also a critical investment in the country’s future prosperity and growth.

## Ethics and consent

Each participating country’s review boards granted ethical approval for the MICS during survey implementation. In Algeria the approval was by the ethics committee from the Algerian Ministry of Health, Population and Hospital Reform – General Directorate of Prevention and Health Promotion according to the Executive Decree No. 92–276 of 06 July 1992 on the Algerian Code of Medical Ethics. The MICS survey included informed consent statements read to respondents who had the option of accepting or declining participation. Verbal consent is typically used to obtain consent in MICS surveys. This method is frequently employed for numerous purposes. Document-Free Process for Sensitive or Personal Topics, Considering Cultural and Literacy Factors.

## Data Availability

The data are available in the public domain and can be accessed through the prescribed registration on the official UNICEF website ( https://mics.unicef.org/surveys) (accessed on 25 Oct 2024).
