# Supplementary material for: Undernutrition before two years in Algeria: Repeated cross-sectional analyses (2012-13 to 2018-19)
Source: F1000Res. 2024 Nov 26;13:1428. [Version 1] doi: 10.12688/f1000research.158413.1 (PMC12579308; doi:10.12688/f1000research.158413.1)
Supplement: Peer review history

# Reviewer report: Reviewer response for version 1

**DOI:** 10.5256/f1000research.173995.r355578

**Published:** 2025-10-31

**Authors:** Asif Khaliq

**Affiliations:** 1 Queensland University of Technology, Queensland, Australia

Abstract: 

 1) I think this sentence is giving same message twice, you need to merge these two sentences. Write prevalence of stunting, wasting and underweight across two survey periods and in the brackets write p-value. Alternatively, only present the stunting prevalence because it is significant. - "Stunting decreased significantly, but wasting and being underweight showed no significant reductions from 2013 to 2019. Stunting, wasting, and underweight decreased by 2.2%, 1.3%, and 0.3%, respectively, with an overall prevalence of 10.1%, 5.4%, and 3.6%, respectively."

 2) Regarding factors there is need to clarify about the causative and protective factors for each form of undernutrition.

 Introduction:

 3) Citation missing - "The World Health Organization (WHO) states that undernutrition accounts for nearly half of all deaths in children under five globally, which could be prevented through maternal and child health interventions." 

 4) Write exact figure rather than using subjective term -"Most undernourished children are in developing countries, with Asian and African countries being the most affected." I found that you presented the prevalence of stunting and wasting in Asia and Africa, you can merge all those sentences and synthesize a new sentence.

 5) Most of the content presented in the background and introduction section of this manuscript is already known. I suggest you remove all those known facts, because it is only increasing word count rather than bringing any new information. See paragraph 3.

 6) Furthermore, I did not find coherence and cohesion in between the sentences and paragraphs. I really like your writing, because it's simple and easy. Better to present your whole background in three paragraphs (1) Global (2) Algeria or Africa (3) Rationale and connect it with first 1000 days of life. 

 Methodology: 

 7) Can you please change the heading study population to study population and their eligibility criteria. Also add a line data of children with valid and complete anthropometry was used for analysis, because we cannot include data of children with incomplete anthropometry or with anthropometric outliers for assessing the nutritional status. 

 8) Remove sub-heading data collection, because the MICS survey has a range of variables and its understandable that you did secondary data analysis. 

 9)  Add citation" "This is represented as a deviation from the median in terms of standard deviations (SDs). Stunting was defined as height-for-age (HAZ) Z-score≤ 2SD;wastingas weight-for-height (WHZ) Z-score≤ 2 SD of the median and underweight as weight-for-age (WAZ) Z-score < 2SDof the median."

 10) How did you deal with data of overweight and obese children, because WAZ and WHZ value when exceeds over +2.00 then it indicates overweight and obesity. 

 11) You cannot keep wealth index and WASH in same equation, because it may leads to colinearity. Even if by chance you are not getting colinear relationship for your data, you need to delete either of these. Reason: Wealth index is a derived variable in MICS and DHS and some of the variables of WASH and household assets were used for calculating wealth index. Owing to that reason, we cannot keep both in same model. I hope this clarifies your confusion. If you keep that then it means you are doing reporting errors.

 12) Remove the second paragraph of potentional confounding factors. Add categories of each study variable in brackets, such as sex (male, female). Also change the subheading title to study covariates.

 13) Add another heading of data management before data analysis. 

 14) Move table 1 in the result section. Within the table 1 add some sub-sections, such as child factor, maternal factor, etc and organise all the variables of each factor within that factor.

 Results:

 15) In the table 1, add one more column and show statistical difference in the distribution of variables across two survey periods. 

 16) Remove all in-text % written inside the brackets NOT from the tables. 

 17) Too much numbers difficult to read the inferential result. I have to remove all the p-value, odds and confidence intervals for reading each sentence. I would suggest you change your result reporting method. You can present odds and p-value, but do not replicate whatever you have presented in tables. Just discuss the significant findings. 

 18) Put ASTERIK sign with the odds and CI rather than prevalence and CI in table 2.

 Discussion: 

 19) No citation in first paragraph.

 20) I really enjoyed reading your discussion part, because it had properly acknowledge other studies from different regions of the world. Almost all the variables discussed appropriately.

Is the work clearly and accurately presented and does it cite the current literature?

Yes

If applicable, is the statistical analysis and its interpretation appropriate?

Yes

Are all the source data underlying the results available to ensure full reproducibility?

Yes

Is the study design appropriate and is the work technically sound?

Yes

Are the conclusions drawn adequately supported by the results?

Yes

Are sufficient details of methods and analysis provided to allow replication by others?

Yes

Reviewer Expertise:

Paediatrics, nutritional disorders, infectious diseases, pharmacovigilence.

I confirm that I have read this submission and believe that I have an appropriate level of expertise to confirm that it is of an acceptable scientific standard.

# Reviewer report: Reviewer response for version 1

**DOI:** 10.5256/f1000research.173995.r355576

**Published:** 2025-01-16

**Authors:** Om Raj Katoch

**Affiliations:** 1 Govt. Maulana Azad Memorial College, Cluster University of Jammu, Jammu & Kashmir, India

The manuscript “ **Undernutrition before two years in Algeria: Repeated cross-sectional analyses (2012-13 to 2018-19)"** offers valuable insights into undernutrition among children in Algeria. However, several areas require clarification and improvement:

1. The methodology section lacks details on the calculation of undernutrition indicators—stunting, wasting, and underweight. Definitions and formulas, such as stunting being a height-for-age Z-score (HAZ) below -2 SD of WHO standards, must be specified. Indicate the software or statistical tools (e.g., WHO Anthro, R, Stata, SPSS) used for analysis.
2. The discussion would benefit from comparing findings with existing literature. The authors should explore contributing factors, such as improved maternal education and healthcare access, and situate these findings within regional trends.
3. The manuscript should explicitly acknowledge the limitations of the study.
4. What are the policy implications of the study to the study are or to the world.

Is the work clearly and accurately presented and does it cite the current literature?

Partly

If applicable, is the statistical analysis and its interpretation appropriate?

Yes

Are all the source data underlying the results available to ensure full reproducibility?

Yes

Is the study design appropriate and is the work technically sound?

Partly

Are the conclusions drawn adequately supported by the results?

Yes

Are sufficient details of methods and analysis provided to allow replication by others?

Partly

Reviewer Expertise:

Economics of Nutrition

I confirm that I have read this submission and believe that I have an appropriate level of expertise to confirm that it is of an acceptable scientific standard.

# Response

**Published:** 2025-01-20

**Authors:** Nelmighrabi Elmighrabi

Dear Om Raj Katoch,

 Thank you for your valuable feedback on my manuscript, “ **Undernutrition before two years in Algeria: Repeated cross-sectional analyses (2012-13 to 2018-19)."** I have carefully considered your comments, and here is a response to each of your comments, outlining the changes made and providing clarification where necessary. I hope these address your concerns and enhance the clarity and quality of the manuscript.

 1. The methodology section lacks details on the calculation of undernutrition indicators—stunting, wasting, and underweight. Definitions and formulas, such as stunting being a height-for-age Z-score (HAZ) below -2 SD of WHO standards, must be specified. Indicate the software or statistical tools (e.g., WHO Anthro, R, Stata, SPSS) used for analysis

**1. Author response:** *The "Outcome Variables" subheading describes three undernutrition indicators—stunting, wasting, and underweight—and their calculation methods.*

*STATA 2017 software was utilised software for the analysis, and we specified that in the data analysis subheading that descriptive analyses were conducted using Stata ‘Svy’ commands (Stata Corp 17.0).*

 2. The discussion would benefit from comparing findings with existing literature. The authors should explore contributing factors, such as improved maternal education and healthcare access, and situate these findings within regional trends.

**2. Author response:** *We strongly agree with this observation. However, due to the limited literature available, to the best of our knowledge, we have addressed this by comparing our results with studies from other countries that share similar circumstances. Specifically, we have included comparisons in nearly every paragraph of the discussion section, such as "The* **findings indicated a significant association between perceived small birth size and a higher likelihood of wasting in children. This finding is consistent with those of a previous study conducted in Nigeria. ^52^ Other studies from Ethiopia, ^53^ Brazil ^54^ and Pakistan ^55", "^The present study found that older children aged 18–23 months were less prone to underweight. The results of this study are consistent with the findings of Eriga et al. and Pheringxay et al., which found that children aged 13-24 months and 12-24 months have a greater chance of being underweight. " ^65, 66^****

*and*

**^"^The current study observed increased odds of stunting and being underweight among mothers with limited education. Similar findings were found in Pakistan, in a sample of 3071 Pakistani children aged 0-59 months from the Pakistan Demographic and Health Surveys (DHS) 2012-2013 found a significant association between undernutrition among children under five and mothers with low education levels. ^43 ^Similarly, a study conducted in Ethiopia aimed to evaluate stunting and its related factors in children aged 6–59 months. ^44^ A systematic study and meta-analysis conducted in middle-income countries also found a statistically significant inverse correlation between maternal educational level and probability of being underweight. ^45^ "**

 - *We also agree with your suggestion regarding the importance of exploring contributing factors. This aspect has been discussed in the manuscript, particularly in the discussion section, where we highlighted healthcare access and maternal education as factors that also associated with undernutrition in Algeria* **. " Children of mothers who did not visit any ANC clinic were significantly more prone to wasting. Previous research demonstrated a significant correlation between mothers’ lack of knowledge of child feeding practices and the occurrence of wasting. ^57^ This corroborates our finding of an association between ANC clinic attendance and wasting, as it is at such places that mothers gain the requisite knowledge of appropriate child-feeding practices, which may be applied to prevent wasting."**

*and*

**"The current study observed increased odds of stunting and being underweight among mothers with limited education. Similar findings were found in Pakistan; a sample of 3071 Pakistani children aged 0-59 months from the Pakistan Demographic and Health Surveys (DHS) 2012-2013 found a significant association between undernutrition among children under five and mothers with low education levels. ^43^ Similarly, a study conducted in Ethiopia aimed to evaluate stunting and its related factors in children aged 6–59 months. ^44^A systematic study and meta-analysis conducted in middle-income countries also found a statistically significant inverse correlation between maternal educational level and probability of being underweight. ^45".^**

 3. The manuscript should explicitly acknowledge the limitations of the study.

***3.*** **Author response:** *The limitations of this study were clearly articulated in the final paragraph of the discussion.*

 4. What are the policy implications of the study to the study are or to the world?

**4**. **Author response: **Policy implications and recommendations have been addressed and stated in the discussion section of the manuscript, specifically before the last two paragraphs that discuss the strengths and limitations of the study.

*Please feel free to share any additional feedback.*

 Best regards,

 Nagwa Elmighrabi
